# Quantification of TNF-*α* in Patients with Periodontitis and Type 2 Diabetes

**DOI:** 10.1155/2019/7984891

**Published:** 2019-07-04

**Authors:** Víctor M. Martínez-Aguilar, Bertha A. Carrillo-Ávila, Eduardo A. Sauri-Esquivel, Eugenia Guzmán-Marín, Matilde Jiménez-Coello, Diana María Escobar-García, Amaury Pozos-Guillén

**Affiliations:** ^1^Department of Specialization in Periodontology, Faculty of Dentistry, Autonomous University of Yucatan, Mérida, YUC 97000, Mexico; ^2^Cellular Biology Laboratory, Regional Research Center “Dr. Hideyo Noguchi”, University of Yucatan, Mérida, YUC 97000, Mexico; ^3^Basic Sciences Laboratory, Faculty of Dentistry, San Luis Potosi University, San Luis Potosí, SLP 78290, Mexico

## Abstract

**Objective:**

The present study aimed to compare variations in quantified tumor necrosis factor-alpha (TNF-*α*) levels in patients with periodontitis stage 2 grade B (POD2B) and/or type 2 diabetes (T2D) and to identify any relationships between this cytokine and these diseases.

**Methods:**

Levels of the cytokine TNF-*α* in gingival crevicular fluid in patients with POD2B and/or T2D were evaluated. A total of 160 subjects were distributed into four groups: those with POD2B (n=44); those with T2D (n=37); those with POD2B/T2D (n=40); and healthy subjects (n=39). Glycosylated hemoglobin (HbA1c) and blood glucose (BG) levels were quantified in each subject. Data were collected on body mass index (BMI), loss of insertion (LI), and probe depth (PD). Gingival crevicular fluid samples were collected from the most acutely affected periodontal pocket and gingival sulcus in each subject, and TNF-*α* was quantified by multiplex analysis.

**Results:**

Kruskal Wallis tests was used to identify differences in TNF-*α* levels, LI, PD, BMI, BG, and HbA1c by group. Differences (p<0.001) were found for LI, PD, BG, and HbA1c. A Spearman test was used to calculate possible correlations between TNF-*α* levels and LI or PD identified a weak but significant negative correlation of TNF-*α* with LI (Rho=-0199; p=0.012), and a moderately positive correlation of LI with PD (Rho=0.509; p < 0.001).

**Conclusions:**

No variation was found between TNF-*α* levels and the presence of POD2B, POD2B/T2D, or T2D, suggesting the absence of any direct relationship between progression of these diseases and TNF-*α* levels. However, a correlation was present between low TNF-*α* concentrations and greater LI.

## 1. Introduction

Diabetes mellitus is a public health problem in many countries. In medical terms, it encompasses a group of metabolic disorders with heterogeneous clinical and genetic characteristics, which manifest as abnormally high blood glucose levels. These disorders have a profound impact on health in affected individuals, causing high morbidity and mortality rates, and constitute an economic burden on health systems [1, 2]. The International Diabetes Federation states that China, India, the United States of America, Brazil, Russia, and Mexico have the highest number of diabetic patients [3]. In 2012, diabetes was estimated to be the direct cause of 1.5 million deaths, and another 2.2 million deaths were attributable to hyperglycemia. Of these 3.7 million deaths, 43% occur in people less than 70 years old. Type 2 diabetes (T2D) accounts for 90 to 95% of cases worldwide and most commonly occurs in adults but is now increasingly common in children. By 2030, diabetes is projected to be the seventh cause of death worldwide [4, 5].

Periodontitis (POD) is the most common form of periodontal disease. It is more prevalent in adults but can occur in children. Although multifactorial in origin, three main factors are involved in POD appearance and evolution: accumulation of plaque and calculus; level of bacterial virulence; and cellular immune response [6]. Evolution is slow to moderate, but more rapid periods of destruction can be observed, influenced mainly by systemic or environmental factors that can affect the normal interaction between host and bacteria [7]. Plaque accumulation and host response to it can be affected by local factors such as systemic diseases (*e.g.,* diabetes mellitus, HIV), which can depress host defenses, and the environment (*e.g.,* smoking and stress) [8].

Both T2D and POD influence oral cavity health. Diabetes is a risk factor for gingivitis and POD when related to formation of a more persistent inflammatory infiltrate. An inverse influence may also exist in that POD could be a risk factor for diabetic decompensation [9]. A complex bidirectional relationship between T2D and POD may occur that would create a vicious circle exacerbating both diseases when present simultaneously in the same individual [10–13].

Recent studies have been focused on the role of cytokines in periodontal diseases in diabetic patients. Cytokines are a group of cell regulators vital in the production and activation of effector cells that initiate and regulate different immune and inflammatory responses [14–16]. Tumor necrosis factor-alpha (TNF-*α*) is a proinflammatory mediator considered to be a soluble mediator released from immunocompetent cells. It exercises a wide range of proinflammatory and immunomodulatory effects in different cell populations, such as stimulating prostaglandin synthesis, promoting tumors in a variety of cancers, producing proteases, and activating osteoclastic function and therefore bone resorption. Its myriad functions suggest that TNF-*α* plays an important role in mediating the immune-inflammatory responses initiated by infection or other types of damage [17]. During its initial production in the inflammatory response, TNF-*α* is also vital for maintaining chronic inflammation, angiogenesis, tissue remodeling, tumor growth, and metastasis; TNF-*α* blockers are therefore effective in treating a variety of acute and chronic inflammatory conditions [18].

An important proinflammatory cytokine and immune response modulator, TNF-*α* production occurs in response to stimuli from cell types such as macrophages, neutrophils, keratinocytes, adipocytes, fibroblasts, and NK, T and B cells. High serum levels of this cytokine have been detected in patients with POD, suggesting it may be contributing to pathogenesis. Its activation also stimulates bone resorption by induction in osteoclast progenitor proliferation, and production of extracellular matrix metalloproteinases, cytokines, collagenase, and prostaglandins [19–22].

The aim of this study was to compare the variations in quantified TNF-*α* levels in patients with POD and/or T2D and to identify any relationships between this cytokine and these diseases.

## 2. Materials and Methods

A cross-sectional study was done and approved by the Institutional Bioethics Committee. Subjects were selected from the Admission Dental Clinic, Faculty of Dentistry, Autonomous University of Yucatan (UADY); after explanation of the procedure, those choosing to participate signed an informed consent.

Presence of T2D was identified based on the 2019 American Diabetes Association parameters [23]: glycosylated hemoglobin (HbA1c) values ≥ 6.5% indicate diabetes and those ≤ 5.6% are normal; glucose blood (GB) levels (8 to 10 hours) ≥ 126 mg/dL indicate diabetes and those ≤ 100 mg/dL are normal.

According to specific classification for periodontal and peri-implant diseases and conditions, patients included were targeted with stage 2, grade B POD [24]. Periodontal probing with a calibrated periodontal probe was done (UNC-15, Hu-Friedy, Chicago, IL, USA); all teeth were examined except third molars. Subjects were excluded who had received periodontal treatment, chemotherapy, and antibiotic and/or anti-inflammatory treatment in the six months prior to examination or exhibited systemic diseases other than T2D. From a total of 160 selected subjects, four study groups were formed: group 1 (POD2B, n=44); group 2 (T2D, n=37); group 3 (POD2B/T2D, n=40); and group 4 (control with healthy subjects exhibiting no periodontal disease, n=39). Gingival crevicular fluid (GCF) samples were collected from periodontal pockets with ≥ 4 mm depth and ≤ 3 mm insertion loss in groups 1 and 3, and from the mesiovestibular gingival sulcus of the first lower molar in groups 2 and 4. Samples were collected by first isolating the tooth with a cotton impeller and removing the supragingival plaque with a curette (Gracey, Hu-Friedy, Chicago, IL, USA), avoiding injury to the marginal gingiva. After slightly drying the crevicular site with air, GCF was obtained by inserting a PerioPaper strip (PerioPaper, ProFlow, Amityville, NY, USA) into the sulcus or periodontal pocket to the point of resistance and leaving it there for thirty seconds. Strips contaminated with saliva or blood were discarded and a new sample taken at a different site. After collection, the PerioPaper strips were immediately placed in sterile Eppendorf vessels and stored at -70°C until analysis. The GCF was extracted by two elution methods in 0.05% PBS-T solution followed by centrifuging at 12,000* g* for 5 minutes and at 4°C, until reaching a final elution volume of 80 *μ*L. Of this volume, 40 *μ*L were tested with Luminex platform (Magpix, Millipore, St. Charles, MO, USA) and analyzed with a MILLIPLEX analyst software (ViageneTech, Carlisle, MA, USA). Results were expressed per mL of elution to measure TNF-*α* levels in the total amount (pg) and concentration volume according to the formula TNF-*α* (pg)/volume (*μ*L).

Kruskal-Wallis test was applied to identify any differences in the data for TNF-*α*, loss of insertion (LI, %), probe depth (PD), body mass index (BMI), blood glucose (BG), and glycosylated hemoglobin (HbA1c) by group. A Spearman test was used to evaluate the possible existence of a correlation between TNF-*α* count and LI or PD (statistical significance p≤0.05).

## 3. Results

Analysis of the BMI, BG, and HbA1c data showed 31.87% of the total sample to exhibit glycemic levels outside normal levels (Table 1). In the comparison between groups, BG and HbA1c had different values (p<0.001). Paired comparisons for BG identified differences (p<0.001) between Control-T2D, Control-POD2B/T2D, POD2B-T2D, and POD2B-POD2B/T2D. For HbA1c, differences (p<0.001) were found between POD2B-T2D, POD2B-POD2B/T2D, Control-T2D, and Control-POD2B /T2D. The T2D and POD2B/T2D groups had the highest values (Table 1).

Analysis of periodontal condition identified differences between groups for LI and PD (p<0.001), with the highest values in both cases being in POD2B and POD2B/T2D (Table 2). No differences in TNF-*α* concentration were found between groups. A Spearman test identified a weak but significant negative correlation between TNF-*α* levels and LI (^*∗∗*^Rho=-0.199; p=0.012), and a moderate but significant positive correlation between LI and PD (^*∗∗∗*^Rho=0.509; p<0.001).

## 4. Discussion

There are multiple conditions that can affect the periodontal health. Locally, factors such as dental malposition, poorly adjusted restorations, maxillofacial fractures, and uncontrolled use of chlorhexidine-based products have been reported [25, 26]. Likewise, several systemic conditions have been reported that influence the periodontal status of patients, among which T2D, hypertension, hemophilia, leukemia, and certain digestive disorders can be mentioned [27–29]. All these conditions have been studied separately, observing important changes in the evolution of periodontitis and the associated immunological factors.

TNF-*α* is an important proinflammatory cytokine and modulator of the immune response. It is produced in response to stimuli from cell types such as macrophages, neutrophils, keratinocytes, adipocytes, fibroblasts, and NK, T and B cells. High serum TNF-*α* levels have been detected in patients with POD since it contributes to disease pathogenesis. This cytokine stimulates bone resorption by inducing osteoclast progenitor proliferation, as well as production of chemokine, extracellular matrix metalloproteinases, cytokines, collagenase, and prostaglandins. Cytokine concentration in GCF has been linked to degree of glycemic control in diabetic patients [19–22].

Studies have shown that diabetic patients with periodontal infection have a higher risk of losing control of their glycemic condition; and this deteriorates over the long term compared to diabetic patients who do not suffer POD [30–32]. In the course of periodontal disease, various proinflammatory mediators occur, such as interleukin- (IL-) 1, IL-6, and IL-8, IFN- *γ*, CCL5, TNF-*α*, prostaglandins, and metalloproteinases. These mediators alter the activity of leukocytes and osteoblasts-osteoclasts and promote the tissue remodeling process both locally and systemically. The proinflammatory cytokine TNF-*α* regulates production of collagenase, prostaglandin E2, molecular adhesion cells, and factors related to bone resorption. Secreted mainly by monocytes and macrophages, elevated TNF-*α* levels have been observed in chronic gingival inflammation processes and in GCF in patients with POD [9, 33, 34].

No intergroup differences in TNF-*α* concentration were observed in the present study. This is similar to the lack of differences in TNF-*α* concentration in the GCF between patients with POD2B, aggressive POD, or systemically healthy patients reported in a Turkish population [35]. However, in the present study, differences were present between groups in terms of LI and PD, with the POD2B and POD2B/T2D groups having the highest values. These results agree with a study of the progression of periodontal lesion in which no differences in TNF-*α* concentration were observed when comparing active and inactive sites in 56 patients in a Chilean population diagnosed with moderate to advanced chronic POD [36, 37]. They also agree with a study done in Brazil in which no differences in TNF-*α* concentration was noted between POD2B/T2D and POD2B patients [38, 39].

Increases in TNF-*α* concentration in patients with POD2B/T2D or POD2B have also been described in a Portuguese population, but with no differences between groups [40, 41], like the present results. A study in Korean patients found no correlation between TNF-*α* levels and gingival tissues in patients with POD [30, 31]. The lack of difference in the present results may have occurred because 68.13% of the patients exhibited good glycemic control. This can translate into low TNF-*α* expression in POD2B/T2D patients because hyperglycemia can overregulate levels of TNF-*α*, and other cytokines such as GM-CSF and IL-6, in both healthy and periodontal affected tissues [42, 43]. This overregulation also enhances epithelial cell stimulation capacity by providing an inflammatory system that must be interrupted for the disease to improve; this interruption can occur when hyperglycemia is controlled or when a periodontal disease enters remission [42].

Reis et al. found no differences in TNF-*α* levels in patients with or without POD2B, and neither did they observe decreases in TNF-*α* levels after nonsurgical periodontal treatment [44]. In another study, quantification of cytokine expression in diseased peri-implant tissue found no differences in TNF-*α* concentrations between sites with mucositis and peri-implantitis in both GCF and saliva [45]. In a comparison of different treatments in residual pockets using the final concentration of acute-phase proteins, no changes were found in TNF-*α* concentration between data from the baseline condition, at 14 days and at 6 months [46].

Duarte et al. found significant differences between TNF-*α* concentration and T2D when comparing healthy and infected sites in patients with and without T2D [40, 47]. This differs from the lack of difference in the present results, perhaps because BG levels in the present study subjects were not very high and 68.13% of the subjects exhibited adequate glycemic control. Expression of TNF-*α* can be attributed to an increase in RAGE or expression of TLR4, which directly affect the response of epithelial cells and their antagonists inducing the proinflammatory cytokine response, as well as an increase in cell surface receiving capacity [48]. These receptors also work collaboratively to induce expression of these cytokines in oral epithelial cells, although this collaboration is not involved in inducing innate immunity receptors. Indeed, a correlation exists between a lack of control of blood sugar levels and deficiency in the epithelial barrier which translates into an increase in expression of the immune receptors of the innate immune response and an exacerbated inflammatory response [49].

Increases in TNF-*α* concentrations have been reported after nonsurgical periodontal treatment, with higher levels in healthy subjects than in unhealthy patients [50]. These results coincide with the present results in which higher TNF-*α* levels were observed in healthy subjects than in the POD2B, POD2B/T2D, and T2D groups. A possible explanation for these reduced inflammatory protein levels in patients with these chronic diseases is that host immune response may be diminished.

Interstudy variation in TNF-*α* concentrations may be due to several factors including periodontal disease severity, subject age, sample type, population type, and technique details such as storage temperature and pretest storage time [51].

GCF has been widely used as a diagnostic tool for various periodontal diseases. Available evidence indicates that GCF can influence the progression of periodontal diseases when combined with systemic diseases, suggesting that local changes in GCF can be reflected as systemic inflammation through direct expression of circulating inflammatory mediators [52]. The presence of T2D plays a fundamental role in development of chronic POD. Its resistance and control can affect TNF-*α* concentrations, possibly explaining the contrasts between different studies mentioned previously.

## 5. Conclusions

The present results indicate that good control of BG in patients with POD2B/T2D can directly influence expression of the TNF-*α* cytokine; however, low TNF-*α* concentrations are directly correlated with greater insertion loss and probe depth.

## Figures and Tables

**Table 1:** General characteristics of sample.

GROUP		BMI	BG^*∗*^	HbA1c^*∗*^
		Mean±SD (Range)	Mean±SD (Range)	Mean±SD (Range)
POD2B	n=44	27.8±3.37 (23.31-38.45)	95.43±10.42 (75-123)	5.61±0.61 (4.7-7.4)
T2D	n=37	29.75±5.18 (21.83-51.78)	150.59±72.66 (78-328)	7.6±1.76 (4.8-11.9)
POD2B /T2D	n=40	29.78±5.05 (20.96-44.85)	201.35±93.00 (68-460)	8.21±1.87 (4.9-12.0)
CONTROL	n=39	28.51±5.44 (20.67-44.27)	95.08±14.02 (75-151)	5.59±0.57 (4.0-6.8)

BMI: Body mass index; BG: Blood glucose; HbA1c: Glycosylated hemoglobin; ^*∗*^p=0.001

**Table 2:** Loss of insertion and probe depth.

GROUP	LI^*∗*,*∗∗*,*∗∗∗*^	PD^*∗*,*∗∗∗*^	TNF-*α*^*∗∗*^
	Mean±SD (Ranges)	Mean±SD (Range)	Mean±SD (Range)
POD2B	n=44	4.68±1.68 (1.66-10.33)	0.48±0.38 (0.10-0.91)
T2D	n=37	2.48±0.48 (1.00-3.00)	0.56±0.38 (0.10-0.91)
POD2B/T2D	n=40	4.55±1.14 (2.66-6.66)	0.50±0.40 (0.10-1.17)
CONTROL	n=39	2.35±0.40 (1.66-3.00)	0.50±0.39 (0.10-1.00)

LI: Loss of insertion; PD: probe depth; Kruskal Wallis test ^*∗*^p=0.001; Spearman test ^*∗∗*,*∗∗∗*^p=0.001

## Data Availability

The general and clinical characteristics of sample used to support the findings of this study are included within the article.
